# *N*-(2,6-Dichloro­phen­yl)-3-methyl­benzamide

**DOI:** 10.1107/S1600536809039889

**Published:** 2009-10-10

**Authors:** B. Thimme Gowda, Miroslav Tokarčík, Jozef Kožíšek, Vinola Zeena Rodrigues, Hartmut Fuess

**Affiliations:** a Department of Chemistry, Mangalore University, Mangalagangotri 574 199, Mangalore, India; b Faculty of Chemical and Food Technology, Slovak Technical University, Radlinského 9, SK-812 37 Bratislava, Slovak Republic; c Institute of Materials Science, Darmstadt University of Technology, Petersenstrasse 23, D-64287 Darmstadt, Germany

## Abstract

In the mol­ecular structure of the title compound, C_14_H_11_Cl_2_NO, the two aromatic rings form a dihedral angle of 70.9 (1)°. The central amido group –NH—C(=O)– makes a dihedral angle of 26.6 (2)° with the methyl­phenyl ring and 82.5 (1)° with the dichloro­phenyl ring. Inter­molecular N—H⋯O hydrogen bonds link the mol­ecules into chains running along the *c* axis of the crystal.

## Related literature

For the preparation of the title compound, see: Gowda *et al.* (2003 ▶). For related structures, see: Bowes *et al.* (2003 ▶); Gowda, Foro *et al.* (2008 ▶); Gowda, Tokarčík *et al.* (2008 ▶); Tokarčík *et al.*, 2009 ▶).
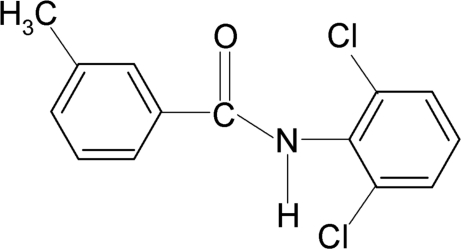


## Experimental

### 

#### Crystal data

C_14_H_11_Cl_2_NO
*M* *_r_* = 280.14
Monoclinic, 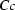

*a* = 11.9433 (8) Å
*b* = 12.5397 (6) Å
*c* = 9.5305 (5) Å
β = 111.859 (7)°
*V* = 1324.72 (13) Å^3^
*Z* = 4
Mo *K*α radiation
μ = 0.48 mm^−1^
*T* = 295 K
0.53 × 0.34 × 0.07 mm

#### Data collection

Oxford Diffraction Xcalibur2 diffractometer with a Sapphire CCD detector
Absorption correction: analytical (CrysAlis Pro; Oxford Diffraction, 2009 ▶) *T*_min_ = 0.756, *T*_max_ = 0.979
28271 measured reflections
2553 independent reflections
2368 reflections with *I* > 2σ(*I*)
*R*_int_ = 0.026

#### Refinement

*R*[*F* ^2^ > 2σ(*F* ^2^)] = 0.028
*wR*(*F* ^2^) = 0.068
*S* = 1.10
2553 reflections
164 parameters
2 restraints
H-atom parameters constrained
Δρ_max_ = 0.18 e Å^−3^
Δρ_min_ = −0.22 e Å^−3^
Absolute structure: Flack (1983 ▶), 1273 Friedel pairs
Flack parameter: −0.02 (5)

### 

Data collection: *CrysAlis Pro* (Oxford Diffraction, 2009 ▶); cell refinement: *CrysAlis Pro*; data reduction: *CrysAlis Pro*; program(s) used to solve structure: *SHELXS97* (Sheldrick, 2008 ▶); program(s) used to refine structure: *SHELXL97* (Sheldrick, 2008 ▶); molecular graphics: *ORTEP-3* (Farrugia, 1997 ▶) and *DIAMOND* (Brandenburg, 2002 ▶); software used to prepare material for publication: *SHELXL97*, *PLATON* (Spek, 2009 ▶) and *WinGX* (Farrugia, 1999 ▶).

## Supplementary Material

Crystal structure: contains datablocks I, global. DOI: 10.1107/S1600536809039889/bt5081sup1.cif

Structure factors: contains datablocks I. DOI: 10.1107/S1600536809039889/bt5081Isup2.hkl

Additional supplementary materials:  crystallographic information; 3D view; checkCIF report

## Figures and Tables

**Table 1:** Hydrogen-bond geometry (Å, °)

*D*—H⋯*A*	*D*—H	H⋯*A*	*D*⋯*A*	*D*—H⋯*A*
N1—H1*N*⋯O1^i^	0.86	2.07	2.866 (2)	155

Symmetry code: (i) 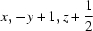.

**Fig. 1.**
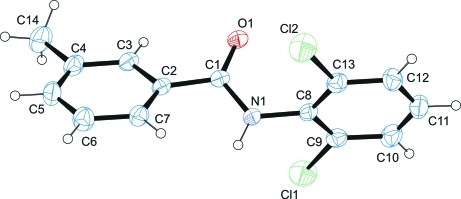
Molecular structure of (I) showing the atom labelling scheme. Displacement ellipsoids are drawn at the 30% probability level. H atoms are represented as small spheres of arbitrary radii.

**Fig. 2.**
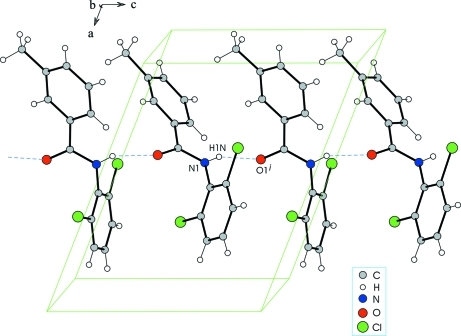
Part of the crystal structure of (I). Molecular chains running along the c-axis are generated by N–H···O(i) hydrogen bonds, shown as dashed lines. Symmetry code (i): x, -y + 1, z + 1/2.

## supplementary crystallographic information

### Comment

As part of a study of the substituent effects on the crystal structures of benzanilides (Gowda, Foro *et al.*, 2008; Gowda, Tokarčík *et al.*, 2008; Tokarčík *et al.*, 2009), in the present work, the structure of *N*-(2,6-dichlorophenyl)-3-methylbenzamide (I) has been determined. The conformations of the N—H and C=O bonds in the amide segment of the structure are anti to each other (Fig.1), similar to that observed in 3-methyl-*N*-(phenyl)benzamide (II) (Gowda, Foro *et al.*, 2008), *N*-(2,6-dichlorophenyl)benzamide (III) (Gowda, Tokarčík *et al.*, 2008), 4-chloro-*N*-(2,6-dichlorophenyl)benzamide (Tokarčík *et al.*, 2009) and the parent benzanilide (Bowes *et al.*, 2003). The central amido group –NH—C(=O)– makes a dihedral angle of 26.6 (2)° with the methyl-phenyl ring and 82.5 (1) ° with the dichloro-phenyl-ring.

The dihedral angle between the two benzene rings in (I) is 70.9 (1)°, compared to the values of of 22.17 (18)°) & 75.86 (12) in the molecules 1 and 2 of (II), respectively, and 56.8 (1)° & 59.1 (1)° in the first and second molecules of (III), respectively. In the crystal structure, the intermolecular N–H···O hydrogen bonds link the molecules into chains running along the *c*-axis of the crystal. (Fig. 2).

### Experimental

The title compound was prepared according to the method described by Gowda *et al.* (2003). The purity of the compound was checked by determining its melting point. It was characterized by recording its infrared and NMR spectra. Colourless single crystals of the title compound were obtained by a slow evaporation from an ethanol solution of the compound (0.5 g in about 30 ml of ethanol) at room temperature.

### Refinement

H atoms were found in difference maps and further treated as riding on their parent atoms, with C–H distances of 0.93 Å (for aromatic C), 0.96 Å (for methyl C) and 0.86 Å (N). The *U*_iso_(H) values were set to 1.2 *U*_eq_(C, N) or 1.5 *U*_eq_(C_methyl_).

### Crystal data

**Table:**

C_14_H_11_Cl_2_NO	*F*(000) = 576
*M**_r_* = 280.14	*D*_x_ = 1.405 Mg m^−^^3^
Monoclinic, *C**c*	Mo *K*α radiation, λ = 0.71073 Å
Hall symbol: C -2yc	Cell parameters from 16815 reflections
*a* = 11.9433 (8) Å	θ = 2.9–29.5°
*b* = 12.5397 (6) Å	µ = 0.48 mm^−^^1^
*c* = 9.5305 (5) Å	*T* = 295 K
β = 111.859 (7)°	Block, colourless
*V* = 1324.72 (13) Å^3^	0.53 × 0.34 × 0.07 mm
*Z* = 4	

### Data collection

**Table:**

Oxford Diffraction Xcalibur2 diffractometer with a Sapphire CCD detector	2553 independent reflections
graphite	2368 reflections with *I* > 2σ(*I*)
Detector resolution: 10.434 pixels mm^-1^	*R*_int_ = 0.026
ω scans	θ_max_ = 25.8°, θ_min_ = 2.9°
Absorption correction: analytical (*CrysAlis PRO*; Oxford Diffraction, 2009)	*h* = −14→14
*T*_min_ = 0.756, *T*_max_ = 0.979	*k* = −15→15
28271 measured reflections	*l* = −11→11

### Refinement

**Table:**

Refinement on *F*^2^	Secondary atom site location: difference Fourier map
Least-squares matrix: full	Hydrogen site location: inferred from neighbouring sites
*R*[*F*^2^ > 2σ(*F*^2^)] = 0.028	H-atom parameters constrained
*wR*(*F*^2^) = 0.068	*w* = 1/[σ^2^(*F*_o_^2^) + (0.0314*P*)^2^ + 0.4349*P*] where *P* = (*F*_o_^2^ + 2*F*_c_^2^)/3
*S* = 1.10	(Δ/σ)_max_ = 0.001
2553 reflections	Δρ_max_ = 0.18 e Å^−^^3^
164 parameters	Δρ_min_ = −0.22 e Å^−^^3^
2 restraints	Absolute structure: Flack (1983), 1272 Friedel pairs
Primary atom site location: structure-invariant direct methods	Flack parameter: −0.02 (5)

### Special details

**Table:**

Geometry. All e.s.d.'s (except the e.s.d. in the dihedral angle between two l.s. planes) are estimated using the full covariance matrix. The cell e.s.d.'s are taken into account individually in the estimation of e.s.d.'s in distances, angles and torsion angles; correlations between e.s.d.'s in cell parameters are only used when they are defined by crystal symmetry. An approximate (isotropic) treatment of cell e.s.d.'s is used for estimating e.s.d.'s involving l.s. planes.
Refinement. Refinement of *F*^2^ against ALL reflections. The weighted *R*-factor *wR* and goodness of fit *S* are based on *F*^2^, conventional *R*-factors *R* are based on *F*, with *F* set to zero for negative *F*^2^. The threshold expression of *F*^2^ > σ(*F*^2^) is used only for calculating *R*-factors(gt) *etc*. and is not relevant to the choice of reflections for refinement. *R*-factors based on *F*^2^ are statistically about twice as large as those based on *F*, and *R*- factors based on ALL data will be even larger.

### Fractional atomic coordinates and isotropic or equivalent isotropic displacement parameters (Å^2^)

**Table:**

	*x*	*y*	*z*	*U*_iso_*/*U*_eq_	
N1	0.47074 (14)	0.47784 (12)	0.42954 (16)	0.0406 (4)	
H1N	0.4424	0.5119	0.4873	0.049*	
C1	0.41176 (16)	0.48266 (14)	0.27880 (19)	0.0359 (4)	
C2	0.30291 (15)	0.55238 (14)	0.22211 (19)	0.0370 (4)	
C3	0.21557 (17)	0.52827 (16)	0.0820 (2)	0.0424 (4)	
H3	0.226	0.469	0.0296	0.051*	
C4	0.11285 (18)	0.59068 (19)	0.0182 (2)	0.0517 (5)	
C5	0.1019 (2)	0.68018 (18)	0.0964 (3)	0.0579 (6)	
H5	0.0352	0.7244	0.0541	0.069*	
C6	0.1874 (2)	0.70552 (18)	0.2356 (2)	0.0569 (5)	
H6	0.1774	0.7657	0.2868	0.068*	
C7	0.28792 (18)	0.64175 (16)	0.2992 (2)	0.0452 (4)	
H7	0.3454	0.6587	0.3935	0.054*	
C8	0.57860 (17)	0.41798 (15)	0.49583 (19)	0.0376 (4)	
C9	0.57762 (19)	0.31764 (15)	0.5562 (2)	0.0455 (4)	
C10	0.6829 (2)	0.26073 (18)	0.6273 (3)	0.0598 (6)	
H10	0.6806	0.1938	0.6682	0.072*	
C11	0.7908 (2)	0.3044 (2)	0.6365 (2)	0.0624 (6)	
H11	0.8621	0.2669	0.6848	0.075*	
C12	0.79466 (19)	0.4020 (2)	0.5755 (2)	0.0564 (5)	
H12	0.8679	0.4304	0.5801	0.068*	
C13	0.68896 (18)	0.45856 (17)	0.5070 (2)	0.0449 (4)	
C14	0.0183 (2)	0.5595 (3)	−0.1330 (3)	0.0798 (8)	
H14A	−0.0283	0.5009	−0.1194	0.12*	
H14B	0.0573	0.5387	−0.2006	0.12*	
H14C	−0.0338	0.6191	−0.175	0.12*	
O1	0.44576 (12)	0.43125 (11)	0.19225 (13)	0.0464 (3)	
Cl1	0.44089 (6)	0.26275 (5)	0.54294 (8)	0.07568 (19)	
Cl2	0.69514 (6)	0.58258 (5)	0.43069 (8)	0.0773 (2)	

### Atomic displacement parameters (Å^2^)

**Table:**

	*U*^11^	*U*^22^	*U*^33^	*U*^12^	*U*^13^	*U*^23^
N1	0.0406 (9)	0.0519 (9)	0.0281 (7)	0.0081 (7)	0.0116 (7)	0.0011 (7)
C1	0.0383 (9)	0.0381 (9)	0.0312 (8)	−0.0051 (7)	0.0128 (8)	−0.0003 (7)
C2	0.0382 (10)	0.0411 (9)	0.0307 (8)	−0.0045 (7)	0.0117 (7)	0.0038 (7)
C3	0.0436 (11)	0.0476 (11)	0.0342 (9)	−0.0031 (8)	0.0125 (8)	0.0028 (8)
C4	0.0399 (11)	0.0677 (14)	0.0398 (10)	−0.0049 (10)	0.0060 (9)	0.0083 (10)
C5	0.0473 (12)	0.0611 (13)	0.0610 (13)	0.0143 (10)	0.0152 (10)	0.0179 (11)
C6	0.0590 (13)	0.0513 (12)	0.0570 (13)	0.0061 (10)	0.0179 (11)	−0.0007 (10)
C7	0.0445 (11)	0.0470 (11)	0.0399 (10)	0.0008 (9)	0.0108 (8)	0.0001 (8)
C8	0.0415 (9)	0.0427 (9)	0.0269 (8)	0.0027 (7)	0.0106 (7)	−0.0029 (7)
C9	0.0526 (11)	0.0471 (10)	0.0384 (9)	−0.0004 (9)	0.0186 (8)	−0.0038 (9)
C10	0.0794 (17)	0.0476 (12)	0.0524 (13)	0.0205 (11)	0.0245 (12)	0.0091 (10)
C11	0.0597 (14)	0.0739 (16)	0.0461 (11)	0.0273 (12)	0.0111 (10)	0.0004 (11)
C12	0.0392 (11)	0.0774 (16)	0.0467 (11)	0.0039 (10)	0.0092 (9)	−0.0051 (11)
C13	0.0457 (11)	0.0504 (11)	0.0351 (9)	−0.0034 (9)	0.0109 (8)	−0.0042 (8)
C14	0.0539 (15)	0.117 (2)	0.0508 (14)	0.0038 (15)	−0.0015 (11)	0.0086 (14)
O1	0.0515 (8)	0.0573 (8)	0.0298 (6)	0.0054 (6)	0.0146 (6)	−0.0021 (6)
Cl1	0.0755 (4)	0.0693 (4)	0.0878 (4)	−0.0162 (3)	0.0367 (3)	0.0088 (3)
Cl2	0.0730 (4)	0.0646 (4)	0.0919 (5)	−0.0154 (3)	0.0281 (3)	0.0157 (3)

### Geometric parameters (Å, °)

**Table:**

N1—C1	1.345 (2)	C7—H7	0.93
N1—C8	1.420 (2)	C8—C13	1.380 (3)
N1—H1N	0.86	C8—C9	1.385 (3)
C1—O1	1.229 (2)	C9—C10	1.384 (3)
C1—C2	1.491 (3)	C9—Cl1	1.733 (2)
C2—C7	1.388 (3)	C10—C11	1.373 (4)
C2—C3	1.388 (3)	C10—H10	0.93
C3—C4	1.390 (3)	C11—C12	1.363 (4)
C3—H3	0.93	C11—H11	0.93
C4—C5	1.380 (3)	C12—C13	1.381 (3)
C4—C14	1.514 (3)	C12—H12	0.93
C5—C6	1.376 (3)	C13—Cl2	1.730 (2)
C5—H5	0.93	C14—H14A	0.96
C6—C7	1.381 (3)	C14—H14B	0.96
C6—H6	0.93	C14—H14C	0.96
			
C1—N1—C8	121.77 (15)	C13—C8—C9	117.29 (18)
C1—N1—H1N	119.1	C13—C8—N1	121.45 (18)
C8—N1—H1N	119.1	C9—C8—N1	121.23 (18)
O1—C1—N1	121.38 (17)	C10—C9—C8	121.7 (2)
O1—C1—C2	121.72 (15)	C10—C9—Cl1	119.38 (16)
N1—C1—C2	116.90 (15)	C8—C9—Cl1	118.94 (16)
C7—C2—C3	119.11 (16)	C11—C10—C9	119.1 (2)
C7—C2—C1	123.28 (15)	C11—C10—H10	120.5
C3—C2—C1	117.55 (16)	C9—C10—H10	120.5
C2—C3—C4	121.54 (18)	C12—C11—C10	120.7 (2)
C2—C3—H3	119.2	C12—C11—H11	119.6
C4—C3—H3	119.2	C10—C11—H11	119.6
C5—C4—C3	117.86 (18)	C11—C12—C13	119.5 (2)
C5—C4—C14	122.5 (2)	C11—C12—H12	120.3
C3—C4—C14	119.6 (2)	C13—C12—H12	120.3
C6—C5—C4	121.51 (19)	C8—C13—C12	121.7 (2)
C6—C5—H5	119.2	C8—C13—Cl2	119.23 (15)
C4—C5—H5	119.2	C12—C13—Cl2	119.04 (17)
C5—C6—C7	120.1 (2)	C4—C14—H14A	109.5
C5—C6—H6	120	C4—C14—H14B	109.5
C7—C6—H6	120	H14A—C14—H14B	109.5
C6—C7—C2	119.84 (17)	C4—C14—H14C	109.5
C6—C7—H7	120.1	H14A—C14—H14C	109.5
C2—C7—H7	120.1	H14B—C14—H14C	109.5
			
C8—N1—C1—O1	3.5 (3)	C1—N1—C8—C13	81.8 (2)
C8—N1—C1—C2	−177.04 (16)	C1—N1—C8—C9	−100.2 (2)
O1—C1—C2—C7	−152.45 (18)	C13—C8—C9—C10	1.1 (3)
N1—C1—C2—C7	28.1 (2)	N1—C8—C9—C10	−176.96 (17)
O1—C1—C2—C3	24.6 (2)	C13—C8—C9—Cl1	−179.28 (13)
N1—C1—C2—C3	−154.84 (16)	N1—C8—C9—Cl1	2.7 (2)
C7—C2—C3—C4	−0.9 (3)	C8—C9—C10—C11	−0.8 (3)
C1—C2—C3—C4	−178.08 (17)	Cl1—C9—C10—C11	179.57 (17)
C2—C3—C4—C5	2.1 (3)	C9—C10—C11—C12	−0.5 (3)
C2—C3—C4—C14	−178.3 (2)	C10—C11—C12—C13	1.5 (3)
C3—C4—C5—C6	−2.2 (3)	C9—C8—C13—C12	−0.1 (3)
C14—C4—C5—C6	178.3 (2)	N1—C8—C13—C12	177.97 (17)
C4—C5—C6—C7	1.0 (4)	C9—C8—C13—Cl2	178.92 (14)
C5—C6—C7—C2	0.4 (3)	N1—C8—C13—Cl2	−3.0 (2)
C3—C2—C7—C6	−0.4 (3)	C11—C12—C13—C8	−1.2 (3)
C1—C2—C7—C6	176.62 (18)	C11—C12—C13—Cl2	179.77 (17)

### Hydrogen-bond geometry (Å, °)

**Table:**

*D*—H···*A*	*D*—H	H···*A*	*D*···*A*	*D*—H···*A*
N1—H1N···O1^i^	0.86	2.07	2.866 (2)	155

Symmetry codes: (i) *x*, −*y*+1, *z*+1/2.
